# More Pathogenicity or Just More Pathogens?—On the Interpretation Problem of Multiple Pathogen Detections with Diagnostic Multiplex Assays

**DOI:** 10.3389/fmicb.2017.01210

**Published:** 2017-06-29

**Authors:** Andreas E. Zautner, Uwe Groß, Matthias F. Emele, Ralf M. Hagen, Hagen Frickmann

**Affiliations:** ^1^Institut für Medizinische Mikrobiologie, Universitätsmedizin GöttingenGöttingen, Germany; ^2^Abteilung A Lehre Gesundheitsversorgung, Sanitätsakademie der BundeswehrMünchen, Germany; ^3^Fachbereich Tropenmedizin am Bernhard-Nocht Institut, Bundeswehrkrankenhaus HamburgHamburg, Germany; ^4^Institut für Medizinische Mikrobiologie, Virologie und Hygiene, Universitätsmedizin RostockRostock, Germany

**Keywords:** multiple detection, facultative pathogenic microorganisms, diagnostics, colonization, infection, molecular diagnostics, PCR

## Abstract

Modern molecular diagnostic approaches in the diagnostic microbiological laboratory like real-time quantitative polymerase chain reaction (qPCR) have led to a considerable increase of diagnostic sensitivity. They usually outperform the diagnostic sensitivity of culture-based approaches. Culture-based diagnostics were found to be insufficiently sensitive for the assessment of the composition of biofilms in chronic wounds and poorly standardized for screenings for enteric colonization with multi-drug resistant bacteria. However, the increased sensitivity of qPCR causes interpretative challenges regarding the attribution of etiological relevance to individual pathogen species in case of multiple detections of facultative pathogenic microorganisms in primarily non-sterile sample materials. This is particularly the case in high-endemicity settings, where continuous exposition to respective microorganisms leads to immunological adaptation and semi-resistance while considerable disease would result in case of exposition of a non-adapted population. While biofilms in chronic wounds show higher pathogenic potential in case of multi-species composition, detection of multiple pathogens in respiratory samples is much more difficult to interpret and asymptomatic enteric colonization with facultative pathogenic microorganisms is frequently observed in high endemicity settings. For respiratory samples and stool samples, cycle-threshold-value-based semi-quantitative interpretation of qPCR results has been suggested. Etiological relevance is assumed if cycle-threshold values are low, suggesting high pathogen loads. Although the procedure is challenged by lacking standardization and methodical issues, first evaluations have led to promising results. Future studies should aim at generally acceptable quantitative cut-off values to allow discrimination of asymptomatic colonization from clinically relevant infection.

## Introduction

The implementation of highly sensitive molecular diagnostic systems in the microbiological diagnostic routine is on the rise. Multiplex polymerase chain reaction (PCR) systems allow the coverage of multiple pathogens in one or several tubes within the same assay and even resistance determinants can be addressed. While increasing standardization and commercialization of such assays allow the reduction of costs and hands-on time, new questions arise regarding the interpretation of the detection of facultative pathogenic microorganisms.

PCR-based assays are usually more expensive but also more sensitive and much faster (**Table 1**) than culture-based methods as shown, e.g., for enteric bacterial pathogens (Wiemer et al., 2011; Polage et al., 2015). This usually increases the quality of diagnosis and offers the possibility of an earlier targeted antimicrobial treatment but may also lead to overdiagnosis, e.g., by the detection of atoxigenic strains, pathogens below the minimum infective dose or the detection of virulence factor genes that are not expressed under the respective conditions.

**Table 1:** Culture-based and culture-independent pathogen detection systems.

Detection system	Sensitivity	Costs	Time to result
**Culture-based pathogen detection**			
Animal culture	Depending on the suitability for the pathogen up to 100%	Varying, usually >$100.00 due to time- and labor-intensiveness	Hours to days
Cell culture	Depending on the suitability for the pathogen up to 100%	Varying, usually $10.00 to $100.00	Days to weeks
Cell-free culture media	Depending on the suitability for the pathogen up to 100%	Usually <$10.00 if commercially available media are used	Usually several days
**Culture-independent pathogen detection**			
Rapid immune-chromatographic antigen-testing	Test-depending, frequently low (70% and less)	Varying, usually about $10.00 per test	Usually an hour or less
Nucleic acid amplification testing (NAT)	Depending on available copy numbers in the sample; detection limits usually between 10 and 10,000 copies per sample	Less than $10.00 in case of in-house systems up to several hundred $ in case of commercial multiplex real-time PCR systems	Less than an hour up to several hours including nucleic acid extraction
Next generation sequencing (NGS)	Depending on available copy numbers in the sample, usually worse than in case of species- or genus-specific NAT	>$1.000 per NGS run	Several days including bio-informatics assessment, less than 2 days in case of automated bio-informatics

The impact of resulting difficult interpretative decisions on the microbiological diagnostic routine, i.e., the interpretation of diagnostic results, is exemplarily summarized in the following.

## Common Sites of Multiple Pathogen Detections and Interpretative Consequences

### Chronic Wounds of Soft Tissue and Skin

The causative effect of human chronic wound microbiota in inducing chronic wound infections was recently demonstrated in line with Koch’s postulates in the murine model (Wolcott et al., 2016), so etiological causality can be considered as confirmed. Further, biofilms in chronic wounds are believed to trigger chronic infections as shown for associations of chronic periodontitis, atherosclerosis, cardiovascular disease, and diabetes (Mihai et al., 2015). Mixed-species biofilms affect wound healing considerably more than mono-species biofilms and the combined pathogenicity predicts the clinical cause (Seth et al., 2012). This synergistic effect of the bacteria within biofilms is triggered by several mechanisms, comprising, e.g., passive resistance, metabolic cooperation, byproduct influence, quorum sensing systems, and an enlarged gene pool with more efficient DNA sharing (Wolcott et al., 2013). Within multi-species infections in chronic wounds, however, biofilm aggregates of low bacterial diversity and even sovereign monospecies biofilms can be observed (Burmølle et al., 2010).

Frequent pathogens, which are isolated from biofilms of chronic wounds by culture-based approaches, comprise *Staphylococcus aureus, Pseudomonas aeruginosa, Enterococcus faecalis*, coagulase-negative staphylococci, Enterobacteriaceae, anaerobic bacteria as well as group G β-hemolytic streptococci (Gjødsbøl et al., 2006; Mihai et al., 2014). Further, *P. aeruginosa* and, to a lower degree, *S. aureus* were shown to form most complex wound biofilms (Mihai et al., 2014). Two or up to five species are detected by bacterial culture in three out of four chronic ulcers (Gjødsbøl et al., 2006). *S. aureus* and *P. aeruginosa* are most frequently detected by culture-based approaches from chronic leg ulcers with comparable detection rates in bioptic samples and swab assessments (Gjødsbøl et al., 2012).

However, molecular approaches allow a deeper insight in the microbial composition of chronic wound infections. Fluorescence *in situ* hybridization was shown to identify *P. aeruginosa* in biofilms in wounds even if culture-based diagnostic approaches failed due to difficult sampling conditions (Kirketerp-Møller et al., 2008). The reason for this failing of culture-based approaches is that the spatial distance of *P. aeruginosa* aggregates from the wound surface is significantly bigger than for aggregates of the more frequently detected *S. aureus* (Fazli et al., 2009). This could also explain the phenomenon of the greater importance of *P. aeruginosa* for keeping chronic wounds arrested in a stage of inflammatory processes (Fazli et al., 2009) and for the larger size of *P. aeruginosa* containing ulcers (Gjødsbøl et al., 2006). This is just one example how culture-based diagnostic approaches systematically underestimate the role of microorganisms in chronic wounds (Wolcott et al., 2016). As shown in animal experiments by next generation sequencing (NGS), facultative pathogenic microorganisms like staphylococci and streptococci, which are typically isolated from wound infections by traditional culture-based approaches, do not necessarily play a quantitatively prominent role in biofilms in case of surgical site infections, while species of Porphyromonadaceae, Deinococcaceae, Methylococcaceae, Nocardiaceae, Alteromonadaceae, and Propionibacteriaceae quantitatively dominated in these assessments (König et al., 2014).

Complex microbial community structure within chronic leg ulcers can be demonstrated in detail by 16S rRNA NGS and quantitative PCR (qPCR; Sprockett et al., 2015), but this approach is still too complex and expensive for the diagnostic routine. While the value of NGS and advanced bioinformatics for the characterization of chronic wounds is widely accepted, their impact on individual therapeutic management decisions which might justify their use in spite of considerable costs is still poorly assessed (Tuttle, 2015).

### The Respiratory Tract

#### Proof of Multiple Respiratory Pathogens—Useful Diagnostic Information or Just More Confusion?

Various multiplex PCR assays are available for the diagnosis of upper respiratory infections and atypical pneumonia (Brittain-Long et al., 2011; Karhu et al., 2014). Interpretative problems can arise from multiple pathogen detections in respiratory specimens. The focus is on the detection of treatable or hygienically relevant viruses such as influenza, adeno, or respiratory syncytial virus (RSV). The reason for screening for other viruses is the intention to reduce unnecessary antibiotic therapy (Oosterheert et al., 2005). Further typical diagnostic indications comprise acute exacerbations of chronic lung diseases like cystic fibrosis (CF) and chronic obstructive pulmonary disease (Frickmann et al., 2012a,b; Markussen et al., 2014).

The upper respiratory tract exhibits a high anatomical complexity with several ecological niches, e.g., the tonsillar crypts or the nasopharynx. Therefore, multiple pathogen detections are frequent in the upper respiratory tract. Multiple pathogens were detected on nasal swabs, in nasopharyngeal aspirates, or in nasal washing specimens of 46% (Martin et al., 2013), 43% (Houben et al., 2010) and 24.5% (Wishaupt et al., 2011) assessed children with respiratory disease, respectively (see **Table 2**).

**Table 2:** Examples of studies demonstrating multiple pathogen detections by PCR in stool and respiratory samples.

Study	Short study description	Number (%) of samples with different pathogens detected per sample
Beckmann et al., 2014	PCR-based screening for 15 enteric pathogens in stool samples of altogether 312 pediatric patients with gastroenteritis and travelers with suspected parasite infestations in Switzerland	8 (2.6%) with two different pathogens
Frickmann et al., 2015a	PCR-based screening for 12 enteric pathogens in 410 stool samples of asymptomatic Madagascan children	5 (1.2%) with four different pathogens, 38 (9.3%) with three different pathogens, 38 (25.9%) with two different pathogens
Frickmann et al., 2015b	PCR-based screening for 17 enteric pathogens in 53 stool samples of European soldiers with diarrhea on deployment in Western African Mali	1 (1.9%) with five different pathogens, 1 (1.9%) with four different pathogens, 9 (17.0%) with three different pathogens, 12 (22.6%) with two different pathogens
Mejia et al., 2013	PCR-based screening for eight enteric parasites in stool samples of four hundred 13-month-old children in rural Ecuador	1 (0.3%) with four different pathogens, 3 (0.8%) with three different pathogens, 30 (7.5%) with two different pathogens
Houben et al., 2010	PCR-based screening for 10 viral and bacterial respiratory pathogens in nasopharyngeal aspirates of 82 infants with episodes of respiratory tract infections in the Netherlands	13 (15.9%) with three different pathogens, 22 (26.8%) with two different pathogens
Martin et al., 2013	PCR-based screening for 15 respiratory viruses and subtypes in nasal swabs in 455 incidents of respiratory illness in 225 childcare-attendees in the USA	4 (0.9%) with five different pathogens, 24 (5.3%) with four different pathogens, 54 (11.9%) with three different pathogens, 130 (28.6%) with two different pathogens
Wishaupt et al., 2011	qPCR-based screening for 17 viral and bacterial pathogens in nasal washing specimens obtained from 583 patients below 12 years of age with suspected acute respiratory infections in the Netherlands	3 (0.5%) with four different pathogens, 23 (4.0%) with three different pathogens, 114 (20.0%) with two different pathogens

Questions arise how to deal with such multiple pathogen detections and how to assign etiological significance. Some authors associate high pathogen load as determined by qPCR with clinical relevance (Houben et al., 2010). However, other factors are likely to play a role as well, comprising first of all clinical symptoms but also the kind of sample material and the assessed anatomical sites. In the exemplary case of co-incident rhinitis, pharyngitis, and bronchitis which may, by chance, be caused by distinct pathogens, a sample from the lower respiratory tract like pharyngeal lavage fluid will contain mechanically spread DNA or RNA of pathogens from all infected sites. In such a case, etiological relevance of different causative agents can exist irrespective of the measured pathogen load.

## The Intestinal Tract

### The Interpretative Problem of Multiple Enteric Pathogen Detections in High Endemicity Settings

In recent years, diagnostic tests for the detection of gastrointestinal pathogens have gradually shifted from microbiological methods like culture, microscopy, and antigen testing to multiplexed DNA-amplification procedures, with increased diagnostic sensitivity (Blessmann et al., 2002; Bruijnesteijn van Coppenraet et al., 2009; Stark et al., 2011; Frickmann et al., 2013; Liu et al., 2014; Verweij, 2014). These molecular methods comprise, e.g., PCR-Luminex, multiplex real-time qPCR, TaqMan array cards, microfluidic qPCR and Lab-on-a-chip devices (Ahmad et al., 2011; Kodani et al., 2011; Czilwik et al., 2015; Tachibana et al., 2015; Tsaloglou et al., 2015). It was shown that the new methods facilitate diagnosis, infection control, and epidemiologic management. In some cases, it is still necessary to supplement them with other methods like resistance testing or bacterial culture (Beckmann et al., 2014). When a positive diagnostic result is obtained, antimicrobial therapy is usually adjusted to the identified pathogen and the associated resistance pattern and a favorable clinical course under appropriate antimicrobial treatment is considered to confirm the diagnostic result.

If, e.g., diagnostic multiplex PCR systems are applied with stool samples from patients usually living under conditions of high hygienic standards in Western industrialized societies, both asymptomatic colonization in apparently healthy individuals and the detection of multiple pathogens in symptomatic patients even in spite of short-term exposition in tropical settings are infrequent (Wiemer et al., 2011; Beckmann et al., 2014). The same applies for apparently healthy soldiers returning from tropical deployments who rarely show chronic infestations with protozoa if appropriate food and water hygiene precautions are enforced (Frickmann et al., 2013). In contrast, high detection rates of facultatively pathogenic microorganisms are observed in stool samples of both symptomatic patients and healthy individuals in settings of high endemicity (Bowen-Jones, 1989; Mejia et al., 2013; Liu et al., 2014; Frickmann et al., 2015b,c; Krumkamp et al., 2015; Eibach et al., 2016).

In such instances, any interpretative discrimination between asymptomatic colonization and clinically relevant infection becomes challenging and requires clinical experience (Platts-Mills et al., 2014; Frickmann et al., 2015b,c). The simultaneous detection of several enteric pathogens in stool samples of people living in high endemic regions impede the etiological attribution and makes an appropriate subsequent therapy, in case of severe diseases, challenging. The high risk of detecting traces of asymptomatic carriage or residual DNA of previous infections (Frickmann et al., 2015c) limits the value of causal attribution of detected pathogens to infectious gastrointestinal disease (see **Table 2**).

Because of this, the importance of the development of evaluated standards for the interpretation of molecular test results from stool samples should be emphasized, both for diagnostic routine and for epidemiological assessments. One option is semi-quantification by counting colony forming units or quantification of pathogen gene equivalents in stool material by qPCR, which is challenging in such complex material due to PCR inhibition (Frickmann et al., 2015c).

In this regard, it is also important to refer to recent studies on colonization resistance. Differences in hygiene, diet, and lifestyle show severe impact on the dissemination of pathogens including the colonization of the gastrointestinal tract. Bereswill and colleagues examined the role of nutrition and obesity with regard to susceptibility to *Campylobacter jejuni* infections in gnotobiotic and obese mice and found out that host-specific intestinal microbiota play a key role in maintaining colonization resistance against *C. jejuni* (Bereswill et al., 2011a,b; Masanta et al., 2013). Therefore, diet and microbiota composition are additional factors that interfere with the decision on etiological relevance.

### Multi-Drug Resistant Bacteria in the Gut—Harmless Colonizers or “Enteric Time-Bomb”?

Exposure to antibiotics crucially contributes to the emergence of resistance, supporting the concept of the resistome, i.e., the collection of all antibiotic resistance genes in a specific niche such as the gut (Schjørring and Krogfelt, 2011; Lawley and Walker, 2013; Chewapreecha, 2014). Recent metagenomic studies have shown an unexpectedly high diversity and abundance in the microbiota of the gut and revealed a close relation to the consumption of antibiotics (Forslund et al., 2013; Hu et al., 2013). Hu et al. (2013) examined the intestinal microbiome of individuals from Denmark, Spain, and China and compared these data to agricultural and environmental data sets. Individuals from China exhibited the highest diversity of resistance genes (70 types of resistance genes in more than 50% of tested individuals) in the gut microbiome, individuals from Denmark the lowest (45 types of resistance genes in more than 50% of tested individuals) (Hu et al., 2013). The observed variations were associated with consumption of antibiotics. High numbers of resistance genes in Chinese individuals coincided with local overuse of antibiotics. The antibiotic resistance genes persisted in the human gut microbiota for a year and more, although such mutations may be accompanied by fitness costs (Forslund et al., 2013; Chewapreecha, 2014).

However, multi-drug resistant (MDR) bacteria in the gut also have the ability to grow on selective media and possibly to overgrow other clinically relevant germs. Due to MDR-resistant Enterobacteriaceae, for example, screening for *Arcobacter* spp. in feces is extremely difficult. In addition, the question of hygiene measures arises in the case of unintended isolation of MDR-resistant Enterobacteriaceae. Over-diagnostics can be reduced by careful pre-analytic decisions on applied diagnostic approaches.

## Methodical Issues

### Bacterial Culture—Screening of Multi-Drug Resistant Bacteria as an Example of Lacking Standardization of Cultural Approaches

Bacterial culture is prone to bias due to multiple colonization and infection. In patients with polymicrobial bacteremia, the risk of dying is virtually identical as in patients with only one detectable pathogen in blood culture (Reuben et al., 1989). Allocation of etiological relevance is difficult in such instances.

In contrast, culture-based identification of certain bacterial pathogens with specific pathogenicity factors or resistance determinants within a complex bacterial colonization flora is even more challenging. This is the case, e.g., for the identification of diarrheagenic *Escherichia coli* among non-pathogenic *E. coli* in stool samples and for the detection of *P. aeruginosa* strains with special resistance determinants in respiratory samples of patients with CF or bronchiectasis, where several strains of the same species are frequently present (Thomassen et al., 1979; Markussen et al., 2014). The usual approach is picking of several colonies for further assessment. However, this is very laborious and may fail if the strain of interest represents only a small proportion of the mixed bacterial flora.

The lack of standardization of screening assays targeting MDR bacteria, that means the lack of optimization and adaptation of diagnostic test assays in a way that they reproducibly lead to the same results with identical samples, is problematic. While swabbing techniques and the kind of used swabs affect the sensitivity of screening for MRSA (Bartolitius et al., 2014; Warnke et al., 2014a,b,c), even basal questions like the need for broth enrichment (Murk et al., 2009; Jazmati et al., 2016), required assessment strategies, and the number of samples (Ho et al., 2012; Vrioni et al., 2012; Rybczynska et al., 2014; Al-Bayssari et al., 2015) are under debate for screening for colonizers of the gut like vancomycin-resistant *Enterococcus* spp. or beta-lactam-resistant Enterobacteriaceae.

Further, detection rates of enteric colonization with extended spectrum beta-lactamase (ESBL) positive Enterobacteriaceae are affected by external factors. Gastrointestinal disorders like travelers’ diarrhea and antibiotic therapies with associated selection pressure were shown to increase the likelihood of identifying enteric colonization with ESBL-positive Enterobacteriaceae in returning travelers (Kantele et al., 2015). Return to Western industrialized countries leads to a decrease of enteric colonization with ESBL-positive Enterobacteriaceae below the detection threshold within months (Ruppe et al., 2012) due to reconstitution of the microbiome of the gut in the absence of interfering factors. In case of persistence in the gut below the detection threshold, screening at hospital admission for pre-existing enteric colonization with resistant bacteria prior to the onset of selecting factors like antibiotic therapy may fail. This makes the definite discrimination of endogenous infections from nosocomial transmissions difficult.

### Molecular Diagnostics—On Interpretative Limitations of Diagnostic PCR

If multiple facultatively pathogenic microorganisms with potential etiological relevance are detected, the attribution of their pathogenic role for the disease of the respective patient is challenging. Quantitative or semi-quantitative approaches in qPCR may overcome this problem, assuming that high pathogen loads are associated with etiological relevance while low quantities indicate mere colonization.

Lindsay et al. (2013) were able to show that high copy numbers (more than 10,000 within the qPCR reaction) of the *ipaH* gene that can be found in case of enteric infections with *Shigella* spp. or enteroinvasive *E. coli* in stool samples are considerably more often associated with clinical disease as well as with cultural confirmation of *Shigella* spp. and blood in stool samples than lower copy numbers. The researchers observed a 5.6-fold increase of diarrhea in cases with more than 29,000 gene copies and suggested a diagnostic cut-off of 14,000 copies.

In a large study comprising 867 stool samples from patients with diarrhea and 619 samples from volunteers without diarrhea that were assessed with three PCR platforms targeting altogether 15 enteric pathogens, Liu et al. (2014) observed higher copy numbers of pathogens in symptomatic than in asymptomatic patients for the most assessed pathogens and test systems. In detail, this quantitative difference was significant for all 15 assessed pathogens using TaqMan array cards, for 14 out of 15 pathogens using a traditional real-time PCR approach (exemption: sapovirus) and for 12 out of 15 pathogens using PCR-Luminex (exemptions: adenovirus, norovirus, *Giardia* spp.).

Houben et al. (2010) reported a negative correlation between the cycle threshold (ct) values of RSV PCR and clinical disease in infants with respiratory tract infections.

Studies by other authors were, however, less convincing. Platts-Mills et al. (2014) detected significantly increased copy numbers of enteric pathogens for rotavirus and *Shigella* spp./enteroinvasive *E. coli* but not for eight other assessed etiologically relevant microorganisms in symptomatic patients. Very low ct-values as low as 14 in qPCR for enteric pathogens were observed in an assessment with stool samples from clinically healthy children in the Madagascan highlands (Frickmann et al., 2015b). In an assessment of European soldiers with diarrhea in Mali, low ct values were observed for different pathogens in some cases of multiple pathogen detections, making any attribution of etiological relevance challenging (Frickmann et al., 2015c).

## Discussion

The combined pathogenicity of multi-species biofilms is believed to maintain the inflammatory activity of chronic infections (Seth et al., 2012). However, for respiratory tract infections and gastrointestinal infections, attribution of etiological relevance is challenging in case of multiple detections of facultatively pathogenic microorganisms by diagnostic molecular multiplex approaches. The use of ct values of real-time PCR for semi-quantification and quantity-based estimation of etiological relevance has been suggested (Houben et al., 2010; Lindsay et al., 2013; Liu et al., 2014). However, only few definite cut-offs for quantitative interpretation have been proposed so far (Lindsay et al., 2013), and studies in high-endemicity settings suggest low ct values even in clinically healthy populations who live under conditions of high colonization pressure (Frickmann et al., 2015b). Next to this, PCR approaches from stool samples are likely to be affected by PCR inhibition (Frickmann et al., 2015a), making ct-value-based semi-quantification difficult. Further, ct-values vary depending on the applied PCR protocol (Frickmann et al., 2015c), so quantitative standards are needed as internal controls. The quantitative interpretation of PCR-based approaches in primarily non-sterile samples materials is—in addition—affected by the detection of remaining nucleic acids from already cleared, previous infections or colonization. Considerable short-term stability of pathogen DNA and even RNA in various biological matrices has been demonstrated recently (Hasan et al., 2012).

Although further standardization is necessary, the quantitative approach is promising. Recently, quantitative NGS with consecutive comparison with negative control samples, which was based on a specific subtraction of reads, was successfully applied for the etiological attribution of Shiga-toxin-producing *E. coli* as the causative agent in 67% stool samples of patients during an outbreak (Loman et al., 2013). Future studies will allow an appropriate standardization of such diagnostic approaches.

## Author Contributions

AZ wrote outline, introduction, abstract, and section on The Respiratory Tract, harmonized all sections. UG wrote outline and section on MDR-bacteria. ME wrote section on The Intestinal Tract. RH wrote section on Chronic Wounds of Soft Tissue and Skin. HF wrote outline, introduction, abstract, and section on Methodical Issues, harmonized all sections.

## Conflict of Interest Statement

The authors declare that the research was conducted in the absence of any commercial or financial relationships that could be construed as a potential conflict of interest.

## References

[B1] Ahmad F. Seyrig G. Tourlousse D. M. Stedtfeld R. D. Tiedje J. M. Hashsham S. A. (2011). A CCD-based fluorescence imaging system for real-time loop-mediated isothermal amplification-based rapid and sensitive detection of waterborne pathogens on microchips. *Biomed. Microdevices* 13 929–937. 10.1007/s10544-011-9562-2 21720851

[B2] Al-Bayssari C. Dabboussi F. Hamze M. Rolain J.-M. (2015). Detection of expanded-spectrum β-lactamases in Gram-negative bacteria in the 21st century. *Expert Rev. Anti Infect. Ther.* 13 1139–1158. 10.1586/14787210.2015.1066247 26162631

[B3] Bartolitius L. Frickmann H. Warnke P. Ottl P. Podbielski A. (2014). Evaluation of an autoclave resistant anatomic nose model for the testing of nasal swabs. *Eur. J. Microbiol. Immunol.* 4 159–165. 10.1556/EUJMI-D-14-00020 PMC4160795 25215192

[B4] Beckmann C. Heininger U. Marti H. Hirsch H. H. (2014). Gastrointestinal pathogens detected by multiplex nucleic acid amplification testing in stools of pediatric patients and patients returning from the tropics. *Infection* 42 961–970. 25015433 10.1007/s15010-014-0656-7

[B5] Bereswill S. Fischer A. Plickert R. Haag L. M. Otto B. Kuhl A. A. (2011a). Novel murine infection models provide deep insights into the “menage a trois” of *Campylobacter jejuni*, microbiota and host innate immunity. *PLoS ONE* 6:e20953 10.1371/journal.pone.0020953 PMC3115961 21698299

[B6] Bereswill S. Plickert R. Fischer A. Kühl A. A. Loddenkemper C. Batra A. (2011b). What you eat is what you get: novel *Campylobacter* models in the quadrangle relationship between nutrition, obesity, microbiota and susceptibility to infection. *Eur. J. Microbiol. Immunol.* 1 237–248. 10.1556/EuJMI.1.2011.3.8 PMC3906620 24516730

[B7] Blessmann J. Buss H. Nu P. A. T. Dinh B. T. Ngo Q. T. V. Van A. L. (2002). Real-time PCR for detection and differentiation of *Entamoeba histolytica* and *Entamoeba dispar* in fecal samples. *J. Clin. Microbiol.* 40 4413–4417. 10.1128/JCM.40.12.4413-4417.2002 12454128 PMC154634

[B8] Bowen-Jones J. (1989). Infection and cross-infection in a paediatric gastro-enteritis unit. *Curationis* 12 30–33. 2561089 10.4102/curationis.v12i3/4.236

[B9] Brittain-Long R. Westin J. Olofsson S. Lindh M. Andersson L. M. (2011). Access to a polymerase chain reaction assay method targeting 13 respiratory viruses can reduce antibiotics: a randomised, controlled trial. *BMC Med.* 9:44 10.1186/1741-7015-9-44 PMC3108322 21521505

[B10] Bruijnesteijn van Coppenraet L. E. Wallinga J. A. Ruijs G. J. Bruins M. J. Verweij J. J. (2009). Parasitological diagnosis combining an internally controlled real-time PCR assay for the detection of four protozoa in stool samples with a testing algorithm for microscopy. *Clin. Microbiol. Infect.* 15 869–874. 10.1111/j.1469-0691.2009.02894.x 19624500

[B11] Burmølle M. Thomsen T. R. Fazli M. Dige I. Christensen L. Homøe P. (2010). Biofilms in chronic infections - a matter of opportunity - monospecies biofilms in multispecies infections. *FEMS Immunol. Med. Microbiol.* 59 324–336. 10.1111/j.1574-695X.2010.00714.x 20602635

[B12] Chewapreecha C. (2014). Your gut microbiota are what you eat. *Nat. Rev. Microbiol.* 12 8 10.1038/nrmicro3186 24336181

[B13] Czilwik G. Messinger T. Strohmeier O. Wadle S. von Stetten F. Paust N. (2015). Rapid and fully automated bacterial pathogen detection on a centrifugal-microfluidic LabDisk using highly sensitive nested PCR with integrated sample preparation. *Lab Chip* 15 3749–3759. 10.1039/c5lc00591d 26235430

[B14] Eibach D. Krumkamp R. Hahn A. Sarpong N. Adu-Sarkodie Y. Leva A. (2016). Application of a multiplex PCR assay for the detection of gastrointestinal pathogens in a rural African setting. *BMC Infect. Dis.* 16:150 10.1186/s12879-016-1481-7 PMC4832549 27080387

[B15] Fazli M. Bjarnsholt T. Kirketerp-Møller K. Jørgensen B. Andersen A. S. Krogfelt K. A. (2009). Nonrandom distribution of *Pseudomonas aeruginosa* and *Staphylococcus aureus* in chronic wounds. *J. Clin. Microbiol.* 47 4084–4089. 10.1128/JCM.01395-09 19812273 PMC2786634

[B16] Forslund K. Sunagawa S. Kultima J. R. Mende D. R. Arumugam M. Typas A. (2013). Country-specific antibiotic use practices impact the human gut resistome. *Genome Res.* 23 1163–1169. 10.1101/gr.155465.113 23568836 PMC3698509

[B17] Frickmann H. Hinz R. Hagen R. M. (2015a). Comparison of an automated nucleic acid extraction system with the column-based procedure. *Eur. J. Microbiol. Immunol.* 5 94–102. 10.1556/EUJMI-D-14-00040 PMC4397851 25883797

[B18] Frickmann H. Jungblut S. Hirche T. O. Groß U. Kuhns M. Zautner A. E. (2012a). Spectrum of viral infections in patients with cystic fibrosis. *Eur. J. Microbiol. Immunol.* 2 161–175. 10.1556/EuJMI.2.2012.3.1 PMC3962751 24688762

[B19] Frickmann H. Jungblut S. Hirche T. O. Groß U. Kuhns M. Zautner A. E. (2012b). The influence of virus infections on the course of COPD. *Eur. J. Microbiol. Immunol.* 2 176–185. 10.1556/EuJMI.2.2012.3.2 PMC3962752 24688763

[B20] Frickmann H. Schwarz N. G. Rakotozandrindrainy R. May J. Hagen R. M. (2015b). PCR for enteric pathogens in high-prevalence settings. What does a positive signal tell us? *Infect. Dis. Lond. Engl.* 47 491–498. 10.3109/23744235.2015.1022212 25761823

[B21] Frickmann H. Schwarz N. G. Wiemer D. F. Fischer M. Tannich E. Scheid P. L. (2013). Food and drinking water hygiene and intestinal protozoa in deployed German soldiers. *Eur. J. Microbiol. Immunol.* 3 53–60. 10.1556/EuJMI.3.2013.1.8 PMC3832084 24265919

[B22] Frickmann H. Warnke P. Frey C. Schmidt S. Janke C. Erkens K. (2015c). Surveillance of food- and smear-transmitted pathogens in European soldiers with diarrhea on deployment in the tropics: experience from the european union training mission (EUTM) Mali. *Biomed. Res. Int.* 2015:e573904 10.1155/2015/573904 PMC4619819 26525953

[B23] Gjødsbøl K. Christensen J. J. Karlsmark T. Jørgensen B. Klein B. M. Krogfelt K. A. (2006). Multiple bacterial species reside in chronic wounds: a longitudinal study. *Int. Wound J.* 3 225–231. 10.1111/j.1742-481X.2006.00159.x 16984578 PMC7951738

[B24] Gjødsbøl K. Skindersoe M. E. Christensen J. J. Karlsmark T. Jørgensen B. Jensen A. M. (2012). No need for biopsies: comparison of three sample techniques for wound microbiota determination. *Int. Wound J.* 9 295–302. 10.1111/j.1742-481X.2011.00883.x 22067000 PMC7950541

[B25] Hasan M. R. Tan R. Al-Rawahi G. N. Thomas E. Tilley P. (2012). Short-term stability of pathogen-specific nucleic acid targets in clinical samples. *J. Clin. Microbiol.* 50 4147–4150. 10.1128/JCM.02659-12 23052319 PMC3502979

[B26] Ho C. Lau A. Cimon K. Farrah K. Gardam M. (2012). *Screening, Isolation, and Decolonization Strategies for Vancomycin-Resistant Enterococci or Extended Spectrum Beta-Lactamase Producing Organisms: A Systematic Review of the Clinical Evidence and Health Services Impact*. Ottawa, ON: Canadian Agency for Drugs and Technologies in Health. 24354039

[B27] Houben M. L. Coenjaerts F. E. J. Rossen J. W. A. Belderbos M. E. Hofland R. W. Kimpen J. L. L. (2010). Disease severity and viral load are correlated in infants with primary respiratory syncytial virus infection in the community. *J. Med. Virol.* 82 1266–1271. 10.1002/jmv.21771 20513094 PMC7167003

[B28] Hu Y. Yang X. Qin J. Lu N. Cheng G. Wu N. (2013). Metagenome-wide analysis of antibiotic resistance genes in a large cohort of human gut microbiota. *Nat. Commun.* 4:2151 10.1038/ncomms3151 23877117

[B29] Jazmati N. Hein R. Hamprecht A. (2016). Use of an enrichment broth improves detection of extended-spectrum-beta-lactamase-producing *Enterobacteriaceae* in clinical stool samples. *J. Clin. Microbiol.* 54 467–470. 10.1128/JCM.02926-15 26607984 PMC4733169

[B30] Kantele A. Lääveri T. Mero S. Vilkman K. Pakkanen S. H. Ollgren J. (2015). Antimicrobials increase travelers’ risk of colonization by extended-spectrum betalactamase-producing *Enterobacteriaceae*. *Clin. Infect. Dis.* 60 837–846. 10.1093/cid/ciu957 25613287 PMC4345818

[B31] Karhu J. Ala-Kokko T. I. Vuorinen T. Ohtonen P. Syrjälä H. (2014). Lower respiratory tract virus findings in mechanically ventilated patients with severe community-acquired pneumonia. *Clin. Infect. Dis.* 59 62–70. 10.1093/cid/ciu237 24729498 PMC4305142

[B32] Kirketerp-Møller K. Jensen P. Ø. Fazli M. Madsen K. G. Pedersen J. Moser C. (2008). Distribution, organization, and ecology of bacteria in chronic wounds. *J. Clin. Microbiol.* 46 2717–2722. 10.1128/JCM.00501-08 18508940 PMC2519454

[B33] Kodani M. Yang G. Conklin L. M. Travis T. C. Whitney C. G. Anderson L. J. (2011). Application of TaqMan low-density arrays for simultaneous detection of multiple respiratory pathogens. *J. Clin. Microbiol.* 49 2175–2182. 10.1128/JCM.02270-10 21471348 PMC3122721

[B34] König L. M. Klopfleisch R. Höper D. Gruber A. D. (2014). Next generation sequencing analysis of biofilms from three dogs with postoperative surgical site infection. *Int. Sch. Res. Notices* 2014:282971 10.1155/2014/282971 PMC4897509 27355023

[B35] Krumkamp R. Sarpong N. Schwarz N. G. Adlkofer J. Adelkofer J. Loag W. (2015). Gastrointestinal infections and diarrheal disease in Ghanaian infants and children: an outpatient case-control study. *PLoS Negl. Trop. Dis.* 9:e0003568 10.1371/journal.pntd.0003568 PMC4349824 25738935

[B36] Lawley T. D. Walker A. W. (2013). Intestinal colonization resistance. *Immunology* 138 1–11. 10.1111/j.1365-2567.2012.03616.x 23240815 PMC3533696

[B37] Lindsay B. Ochieng J. B. Ikumapayi U. N. Toure A. Ahmed D. Li S. (2013). Quantitative PCR for detection of *Shigella* improves ascertainment of *Shigella* burden in children with moderate-to-severe diarrhea in low-income countries. *J. Clin. Microbiol.* 51 1740–1746. 10.1128/JCM.02713-12 23536399 PMC3716050

[B38] Liu J. Kabir F. Manneh J. Lertsethtakarn P. Begum S. Gratz J. (2014). Development and assessment of molecular diagnostic tests for 15 enteropathogens causing childhood diarrhoea: a multicentre study. *Lancet Infect. Dis.* 14 716–724. 10.1016/S1473-3099(14)70808-4 25022434

[B39] Loman N. J. Constantinidou C. Christner M. Rohde H. Chan J. Z.-M. Quick J. (2013). A culture-independent sequence-based metagenomics approach to the investigation of an outbreak of Shiga-toxigenic *Escherichia coli* O104:H4. *JAMA* 309 1502–1510. 10.1001/jama.2013.3231 23571589

[B40] Markussen T. Marvig R. L. Gómez-Lozano M. Aanæs K. Burleigh A. E. Høiby N. (2014). Environmental heterogeneity drives within-host diversification and evolution of *Pseudomonas aeruginosa*. *mBio* 5:e01592-14 10.1128/mBio.01592-14 PMC4172072 25227464

[B41] Martin E. T. Fairchok M. P. Stednick Z. J. Kuypers J. Englund J. A. (2013). Epidemiology of multiple respiratory viruses in childcare attendees. *J. Infect. Dis.* 207 982–989. 10.1093/infdis/jis934 23288925 PMC7107308

[B42] Masanta W. O. Heimesaat M. M. Bereswill S. Tareen A. M. Lugert R. Groß U. (2013). Modification of intestinal microbiota and its consequences for innate immune response in the pathogenesis of Campylobacteriosis. *Clin. Dev. Immunol.* 2013:e526860 10.1155/2013/526860 PMC3845433 24324507

[B43] Mejia R. Vicuña Y. Broncano N. Sandoval C. Vaca M. Chico M. (2013). A novel, multi-parallel, real-time polymerase chain reaction approach for eight gastrointestinal parasites provides improved diagnostic capabilities to resource-limited at-risk populations. *Am. J. Trop. Med. Hyg.* 88 1041–1047. 10.4269/ajtmh.12-0726 23509117 PMC3752800

[B44] Mihai M. M. Holban A. M. Giurcăneanu C. Popa L. G. Buzea M. Filipov M. (2014). Identification and phenotypic characterization of the most frequent bacterial etiologies in chronic skin ulcers. *Rom. J. Morphol. Embryol.* 55 1401–1408. 25611273

[B45] Mihai M. M. Holban A. M. Giurcaneanu C. Popa L. G. Oanea R. M. Lazar V. (2015). Microbial biofilms: impact on the pathogenesis of periodontitis, cystic fibrosis, chronic wounds and medical device-related infections. *Curr. Top. Med. Chem.* 15 1552–1576. 25877092 10.2174/1568026615666150414123800

[B46] Murk J.-L. A. N. Heddema E. R. Hess D. L. J. Bogaards J. A. Vandenbroucke-Grauls C. M. J. E. Debets-Ossenkopp Y. J. (2009). Enrichment broth improved detection of extended-spectrum-beta-lactamase-producing bacteria in throat and rectal surveillance cultures of samples from patients in intensive care units. *J. Clin. Microbiol.* 47 1885–1887. 10.1128/JCM.01406-08 19386837 PMC2691084

[B47] Oosterheert J. J. van Loon A. M. Schuurman R. Hoepelman A. I. Hak E. Thijsen S. (2005). Impact of rapid detection of viral and atypical bacterial pathogens by real-time polymerase chain reaction for patients with lower respiratory tract infection. *Clin. Infect. Dis.* 41 1438–1444. 10.1086/497134 16231254 PMC7107964

[B48] Platts-Mills J. A. Gratz J. Mduma E. Svensen E. Amour C. Liu J. (2014). Association between stool enteropathogen quantity and disease in Tanzanian children using Taqman array cards: a nested case-control study. *Am. J. Trop. Med. Hyg.* 90 133–138. 10.4269/ajtmh.13-0439 24189366 PMC3886409

[B49] Polage C. R. Gyorke C. E. Kennedy M. A. Leslie J. L. Chin D. L. Wang S. (2015). Overdiagnosis of *Clostridium difficile* infection in the molecular test era. *JAMA Intern. Med.* 175 1792–1801. 10.1001/jamainternmed.2015.4114 26348734 PMC4948649

[B50] Reuben A. G. Musher D. M. Hamill R. J. Broucke I. (1989). Polymicrobial bacteremia: clinical and microbiologic patterns. *Rev. Infect. Dis.* 11 161–183. 2649955 10.1093/clinids/11.2.161

[B51] Ruppe E. Pitsch A. Tubach F. de Lastours V. Chau F. Pasquet B. (2012). Clinical predictive values of extended-spectrum beta-lactamase carriage in patients admitted to medical wards. *Eur. J. Clin. Microbiol. Infect. Dis.* 31 319–325. 10.1007/s10096-011-1313-z 21660500

[B52] Rybczynska H. Melander E. Johansson H. Lundberg F. (2014). Efficacy of a once-a-week screening programme to control extended-spectrum beta-lactamase-producing bacteria in a neonatal intensive care unit. *Scand. J. Infect. Dis.* 46 426–432. 10.3109/00365548.2014.896027 24689959

[B53] Schjørring S. Krogfelt K. A. (2011). Assessment of bacterial antibiotic resistance transfer in the gut. *Int. J. Microbiol.* 2011:e312956 10.1155/2011/312956 PMC3034945 21318188

[B54] Seth A. K. Geringer M. R. Hong S. J. Leung K. P. Galiano R. D. Mustoe T. A. (2012). Comparative analysis of single-species and polybacterial wound biofilms using a quantitative. In vivo, rabbit ear model. *PLOS ONE* 7:e42897 10.1371/journal.pone.0042897 PMC3414496 22905182

[B55] Sprockett D. D. Ammons C. G. Tuttle M. S. (2015). Use of 16S rRNA sequencing and quantitative PCR to correlate venous leg ulcer bacterial bioburden dynamics with wound expansion, antibiotic therapy, and healing. *Wound Repair Regen.* 23 765–771. 10.1111/wrr.12309 25902876 PMC5007860

[B56] Stark D. Al-Qassab S. E. Barratt J. L. N. Stanley K. Roberts T. Marriott D. (2011). Evaluation of multiplex tandem real-time PCR for detection of *Cryptosporidium* spp., *Dientamoeba fragilis, Entamoeba histolytica*, and *Giardia intestinalis* in clinical stool samples. *J. Clin. Microbiol.* 49 257–262. 10.1128/JCM.01796-10 21048004 PMC3020426

[B57] Tachibana H. Saito M. Shibuya S. Tsuji K. Miyagawa N. Yamanaka K. (2015). On-chip quantitative detection of pathogen genes by autonomous microfluidic PCR platform. *Biosens. Bioelectron.* 74 725–730. 10.1016/j.bios.2015.07.009 26210470

[B58] Thomassen M. J. Demko C. A. Boxerbaum B. Stern R. C. Kuchenbrod P. J. (1979). Multiple of isolates of *Pseudomonas aeruginosa* with differing antimicrobial susceptibility patterns from patients with cystic fibrosis. *J. Infect. Dis.* 140 873–880. 120383 10.1093/infdis/140.6.873

[B59] Tsaloglou M.-N. Watson R. J. Rushworth C. M. Zhao Y. Niu X. Sutton J. M. (2015). Real-time microfluidic recombinase polymerase amplification for the toxin B gene of *Clostridium difficile* on a SlipChip platform. *Analyst* 140 258–264. 10.1039/c4an01683a 25371968

[B60] Tuttle M. S. (2015). Association between microbial bioburden and healing outcomes in venous leg ulcers: a review of the evidence. *Adv. Wound Care* 4 1–11. 10.1089/wound.2014.0535 PMC4281836 25566410

[B61] Verweij J. J. (2014). Application of PCR-based methods for diagnosis of intestinal parasitic infections in the clinical laboratory. *Parasitology* 141 1863–1872. 10.1017/S0031182014000419 24780241

[B62] Vrioni G. Daniil I. Voulgari E. Ranellou K. Koumaki V. Ghirardi S. (2012). Comparative evaluation of a prototype chromogenic medium (ChromID CARBA) for detecting carbapenemase-producing *Enterobacteriaceae* in surveillance rectal swabs. *J. Clin. Microbiol.* 50 1841–1846. 10.1128/JCM.06848-11 22461675 PMC3372115

[B63] Warnke P. Frickmann H. Ottl P. Podbielski A. (2014a). Nasal screening for MRSA: different swabs – different results! *PLOS ONE* 9:e111627 10.1371/journal.pone.0111627 PMC4213029 25353631

[B64] Warnke P. Harnack T. Ottl P. Kundt G. Podbielski A. (2014b). Nasal screening for *Staphylococcus aureus*–daily routine with improvement potentials. *PLoS ONE* 9:e89667 10.1371/journal.pone.0089667 PMC3933644 24586949

[B65] Warnke P. Warning L. Podbielski A. (2014c). Some are more equal–a comparative study on swab uptake and release of bacterial suspensions. *PLoS ONE* 9:e102215 10.1371/journal.pone.0102215 PMC4092111 25010422

[B66] Wiemer D. Loderstaedt U. von Wulffen H. Priesnitz S. Fischer M. Tannich E. (2011). Real-time multiplex PCR for simultaneous detection of *Campylobacter jejuni, Salmonella, Shigella* and *Yersinia* species in fecal samples. *Int. J. Med. Microbiol.* 301 577–584. 10.1016/j.ijmm.2011.06.001 21855409

[B67] Wishaupt J. O. Russcher A. Smeets L. C. Versteegh F. G. A. Hartwig N. G. (2011). Clinical impact of RT-PCR for pediatric acute respiratory infections: a controlled clinical trial. *Pediatrics* 128 e1113–e1120. 10.1542/peds.2010-2779 21987698

[B68] Wolcott R. Costerton J. W. Raoult D. Cutler S. J. (2013). The polymicrobial nature of biofilm infection. *Clin. Microbiol. Infect.* 19 107–112. 10.1111/j.1469-0691.2012.04001.x 22925473

[B69] Wolcott R. Sanford N. Gabrilska R. Oates J. L. Wilkinson J. E. Rumbaugh K. P. (2016). Microbiota is a primary cause of pathogenesis of chronic wounds. *J. Wound Care* 25 S33–S43. 10.12968/jowc.2016.25.Sup10.S33 27681809

